# Sodium citrate supplementation enhances tennis skill performance: a crossover, placebo-controlled, double blind study

**DOI:** 10.1186/s12970-019-0297-4

**Published:** 2019-08-01

**Authors:** Vivian C. R. Cunha, Marcelo S. Aoki, Michael C. Zourdos, Rodrigo V. Gomes, Wesley P. Barbosa, Marcelo Massa, Alexandre Moreira, Caroline D. Capitani

**Affiliations:** 1 School of Applied Sciences, University of Campinas, Rua Pedro Zaccaria 1300, Limeira, 13484-350 Sao Paulo Brazil; 2 School of Arts, Sciences and Humanities, University of Sao Paulo, Sao Paulo, Sao Paulo Brazil; 3 Department of Exercise Science and Health Promotion, Muscle Physiology Laboratory, Florida Atlantic University, Kissimme, Florida USA; 4 School of Physical Education and Sport, University of Sao Paulo, Sao Paulo, Sao Paulo Brazil

**Keywords:** Buffering agent, Fatigue, Tennis skills, Blood lactate, Supplementation

## Abstract

**Background:**

The efficacy of sodium citrate supplementation (SC) in exercise performance is unclear. Therefore, the aim of this study was to investigate the effect of SC on skilled tennis performance.

**Methods:**

Ten Brazilian nationally-ranked young male tennis players (age: 17 ± 1 yrs.; stature: 176.7 ± 5.2 cm; body mass: 68.4 ± 7.9 kg) participated in this crossover, placebo-controlled, double-blind study. Upon arrival, at baseline, in both experimental sessions blood was collected, then subjects ingested either sodium citrate (SC - 0.5 g^.^kg^−1^BM in capsules of 500 mg) or a placebo (PLA). Two hours later, pre-match blood was collected then skills tests (skill tennis performance test - STPT, repeated-sprint ability shuttle test - RSA) were performed followed by a 1-h simulated match. Immediately following the match, blood was again collected, and STPT, and RSA were administered.

**Results:**

All metabolic parameters (i.e. base excess, pH, bicarbonate, and blood lactate) increased (*p* < 0.001) from baseline to pre-match and post-match in SC condition. Each metabolic parameter was greater (p < 0.001) in SC compared to PLA condition at both pre- and post-match. The SC condition elicited a greater (*p* < 0.01) shot consistency at post-match in the STPT vs. PLA condition (SC: 58.5 ± 14.8% vs. PLA: 40.4 ± 10.4%). A greater (*p* < 0.001) amount of games won was observed in the simulated match for SC condition vs. PLA condition (SC: 8.0 ± 1.6 vs. PLA: 6.0 ± 1.7). Additionally, the games won during the simulated match in SC condition was positively correlated with percentage shot consistency (r = 0.67, *p* < 0.001).

**Conclusions:**

The current findings suggest that SC supplementation is an effective ergogenic aid to enhance skilled tennis performance.

## Introduction

Tennis match play consists of intense intermittent activity and potentially long duration (i.e. up to 5 h); thus, the magnitude of physiological demands during match play can be considerable. Although, blood lactate concentration ([La]) usually remains lower than 5 mmol^.^L^− 1^ [1–4] data have reported [La] concentration to reach 7-8 mmol.L^− 1^ during match play, which has led to both technical and tactical performance declines [5]. The rise in [La] is a consequence of the ≈1:2 work-to-rest ratio during a match [2, 5–7]. Correspondingly, HR consistently reaches 60–80% of maximum with absolute values up to 200 bpm [2] along with VO_2_ rising to 60–70% of maximum [8]. Additionally, the elevation of cortisol [9] and creatine kinase [9, 10] following match play signifies both acute stress and muscle damage.

Although various forms of fatigue exist during tennis match play [11], peripheral fatigue due to diminished ATPase activity compromises muscle contraction capabilities [12]. Further, debate exists regarding if an increase in H^+^ causing metabolic acidosis (i.e. decreased muscle pH) harms [13, 14], helps [15], or has a neutral performance effect [16]. Controversy is also present regarding the influence of the allocation of other ions (Ca^2+^, K^+^, and CL^−^) [17] in addition to decreased pH on exercise performance. However, in tennis, the muscle damage associated with the intermittent nature of tennis may lead to decreased Ca^2+^ release from the sarcoplasmic reticulum negatively affecting force production capabilities, and possibly lead to mistimed strokes [18]. Therefore, strategies, such as nutritional supplementation, which may delay these mechanism of fatigue during match play warrant investigation.

Specifically, extracellular buffers, sodium bicarbonate (NaHCO_3_) and sodium citrate (SC), serve as alkalizing agents and can elicit acute performance benefits in intermittent-type exercise [8, 17, 19–22]. Specifically, data have demonstrated NaHCO_3_ to significantly improve exercise performance, which lasts between 1 and 7 min. [19, 20] and meta-analytics have determined a moderate effect size between NaHCO_3_ and acute exercise performance [21]. Indeed, Wu et al. [23] demonstrated NaHCO_3_ to improve skilled tennis performance following a simulated match, which was associated with increased pH. However, an important limitation of NaHCO_3_ supplementation is that ingestion has consistently resulted in gastrointestinal (GI) distress and even vomiting [24], which may preclude some individuals from experiencing performance benefits [25]. On the other hand, SC supplementation may provide similar benefits to NaHCO_3_ with less GI distress [26].

Similar to NaHCO_3_, SC directly improves extracellular buffering capacity along with indirectly enhancing intramuscular pH conditions via facilitation of H^+^ efflux [22]_._ Specifically, SC supplementation causes the negatively charged citrate anion to be ejected from the plasma leading to decreased plasma H^+^ along with a concomitant increase in bicarbonate (HCO_3_^−^) [8], subsequently improving buffering capabilities. Conflicting results exist with SC as 0.5 g^.^kg^−1^BM (body mass) has been shown to improve anaerobic cycling power [27] performance [28], and has enhanced 200 m swim performance [29]. However, SC failed to improve 5.000 m treadmill running and repeated 60s sprint performance [30]. A meta-analysis from Carr et al. [31] revealed an unclear effect for SC to improve exercise performance, however, limited data was available for analysis and confidence limits were wide [31]. Moreover, Carr et al. [31] did report that SC provided similar benefits to pre-exercise alkalosis as NaHCO_3_ and did note that GI disturbance should be taken into account when considering an alkalizing agent. Despite the potential benefits for SC and tennis performance, to the authors’ knowledge, SC has not yet been examined in this regard.

Therefore, the primary aim of this study was to investigate the effects of SC supplementation (vs. a placebo condition) on specific tennis skill tests in nationally ranked young male tennis players. It was hypothesized that SC would create a condition of metabolic alkalosis; thus, preventing decline in tennis skill performance compared to a condition.

## Methods

### Experimental approach to the problem

This study used a randomized crossover, placebo-controlled, double blind design. Each subject reported for data collection on 3 occasions. The first and second sessions were separated by 3 days and the second and third were separated by 10 days. The first visit served to familiarize subjects with the skill tennis performance test (STPT) and repeated-sprint ability shuttle test (RSA). The second and third visits served as experimental sessions to examine the effects of SC (vs. a placebo-PLA condition) on 4 metabolic parameters (1. base excess - BE, 2. pH, 3. HCO_3_^−^, and 4. [La]), 2 performance tests (i.e. STPT and RSA) and session rating of perceived exertion (RPE) following 1-h of simulated match play. The 10-day period between sessions served as a washout period. All sessions were performed on the same outdoor hard-surface tennis court at 9:30 AM. Environmental conditions, temperature and humidity, were similar between sessions (Second visit: Temperature: 27 ± 2 °C; Humidity: 68 ± 4%; Third visit: Temperature: 26 ± 3 °C; Humidity: 72 ± 3%). Additionally, players were asked to maintain their regular dietary intake (24 h prior to each visit) and their current training program between laboratory visits. Athletes were also instructed to keep their regular diet throughout the duration of the study.

### Subjects

Ten young male tennis players (age: 17 ± 1 years; stature: 176.7 ± 5.2 cm; body mass: 68.4 ± 7.9 kg; body fat: 11.7 ± 1.4%) who were competitive at the Brazilian national level participated in this study. Body fat was estimated by skinfold thickness measurements using a skinfold caliper (Lange, USA). The measurements were taken from the subjects’ right side at the following 7 sites: subscapular, triceps, pectoral, mid-axillary, supra-iliac, abdominal, and anterior mid-thigh. Body density was estimated using the formula proposed by Jackson and Pollock (1978) [32], and body fat was calculated with the equation from Siri [33]. These players were all ranked between 10 and 55 nationally and volunteered for the study. Additionally, all subjects had a training experience of at least 5 years and were currently engaged in training volume of 25–30 h per week. To fully inform the athletes of the protocol the investigators explained the experimental procedures and any potential risks to all subjects and their parents prior to participation. Written informed consent was obtained from the players and their parents. The protocol was approved by the University’s Ethics Committee (protocol #217.695, UNICAMP).

### Experimental protocol

In the experimental sessions, subjects received a standardized meal (shake containing carbohydrates 2 g^.^kg^−1^BM and whey protein 1 g^.^kg^−1^BM) (07:00 AM). At the tennis academy (7:30 AM), 1 mL of blood was collected from subjects’ antecubital vein using a syringe without anticoagulant. Next, either SC in the amount of 0.5 g^.^kg^−1^BM in capsules of 500 mg [8, 29, 31] or a placebo consisting of NaCl 0.1 g^.^kg^−1^BM, microcrystalline cellulose powder 0.3 g^.^kg^−1^BM, and monohydrate lactose 0.1 g^.^kg^−1^BM in capsules of 500 mg were ingested with 1 L of water. Players were instructed to finish the entire 1 L of water within 2 h, which was just before the next blood collection. All capsules were prepared and distributed to subjects by a pharmacist. Following SC or placebo ingestion, players rested for 2 h before a pre-match blood collection in the amount of 1 mL. Immediately following this blood collection, the players then underwent the STPT and RSA tests and then participated in 1-h of simulated match play as outlined by Gomes et al. [34]. Kilit et al. [35] reported similar HR [5] along with higher RPE in service vs. return games [36, 37] in a 1-h simulated match, which is consistent with data observed in actual match play. The simulated match was carried out according to official international tennis federation rules, qualified umpires kept the match score, and matches took place on a clay court. At the conclusion of match play (post-match) a final 1 mL blood collection was conducted and players then performed a post-match STPT and RSA tests. Thirty minutes following the simulated match, players recorded a session RPE value and completed a GI distress questionnaire [38]. Finally, in the simulated match, the coaches paired players against each other according to skill level. All players faced the same opponent in both conditions.

### Physical performance tests

#### Skill tennis performance test (STPT)

This test, previously used [39] assessed fatigue via measurements of stroke accuracy (%), stroke consistency (%), and number of strokes. To perform this test, tennis players received a random ball-feed of 30 balls per minute (1 ball every 2 s) from a tennis ball-serving machine (Lobmaster Poplob™). Balls were fed at a 10^°^ projection with a constant initial velocity of 26.8 m∙s^− 1^ or 96.6 km∙h^− 1^) and landed 2 m in front of the baseline. Determination of fatigue was when the hitting frequency could not be maintained for 2 consecutive ball feeds or when a player voluntarily chose to cease the test. Further, tennis players were instructed to return the ball using the same hitting style, spin, and pace for all shots, and hit each shot at maximal effort. Players were also instructed to aim the shot to land within the singles court but beyond the service line and toward 1 of 2 targets placed in the opposite court. All shots were recorded with video so that analysis could determine stroke accuracy (%), stroke consistency (%), and total number of strokes during the test.

#### Repeated-Sprint ability shuttle test (RSA)

The RSA has been previously utilized to in part determine profile of a tennis player [52]. To measure RSA, a total of 10 shuttle sprints each totaling 22 m were performed as previously outlined by Fernandez-Fernandez et al. [40]. To begin, tennis players stood with his racket in the front position and in the middle of the baseline, while looking at the net. In response to a signal from the investigator players turned and ran to the prescribed backhand (left) or forehand (right) corner. Next, players ran toward the prescribed corner forward and in a straight line and when their feet were in line with a turning point (marked with a cone) players touched the cone with their racket and turned 180^°^. Then, the players ran back to the opposite side of the court while running forward to touch a second cone, and finally turned at that cone to run back to the initial starting position. Following the completion of the RSA trial, players had 15 s of passive recovery before completing the subsequent trial and this continued until all 10 trials were complete. Additionally, the tennis players assumed the ready position 5 s before each trial and waited for the investigator’s signal. The mean time of 10 trials was used for analysis, and the change in time from the first to 10th sprint was calculated in each condition.

#### Rating of perceived exertion (RPE)

Session RPE was recorded to gauge intensity and fatigue of the total session (simulated match, STPT, and RSA) using the CR-10 RPE scale as previously explained by Foster [41]. To assess exertion of the entire session RPE was collected 30 min following completion of the post-match STPT and RSA and the players were individually asked, “How was your workout?” and shown the RPE scale. The same investigator collected all RPE data; and players were familiarized with the RPE scale during the initial visit.

### Metabolic parameters and blood analysis

The 1 mL sample of the metabolic parameters (BE, pH, HCO_3_^−^, and [La]) was analyzed via the iSTAT Blood Gas Analyzer using the disposable cartridge (CG8+) (iSTAT© Abbott, USA).

### Statistical analyses

Means and standard deviations were calculated for all dependent variables at all time points and a Shapiro-wilk test was used to test normality of the data. Metabolic parameters as well as percentage of stroke accuracy and consistency during the STPT were analyzed using a 2-way repeated measures analysis of variance (ANOVA). In the event of a significant F-ratio, a Tukey post hoc test was performed for pairwise comparisons. Number of total strokes during the STPT, mean RSA test time, and games won during the simulated match were analyzed by a paired t-test. Additionally, a linear regression was utilized to determine if any correlation existed between changes in any of the metabolic parameters or STPT with games won during the simulated match. In all cases the level of significance was set at *p* ≤ 0.05. Correlations were interpreted and reported as “weak” if they were less than or equal to 0.35, “moderate” if they fell between 0.36 to 0.67, “strong” if they fell between 0.68 to 0.89, and “very strong” if they were equal or greater than 0.90 [42]. Finally, effect size (ES) was determined using Cohen’s *d* and interpreted in accordance with Cohen (1988) [43].

## Results

### Physical performance tests

**Fig. 1:**
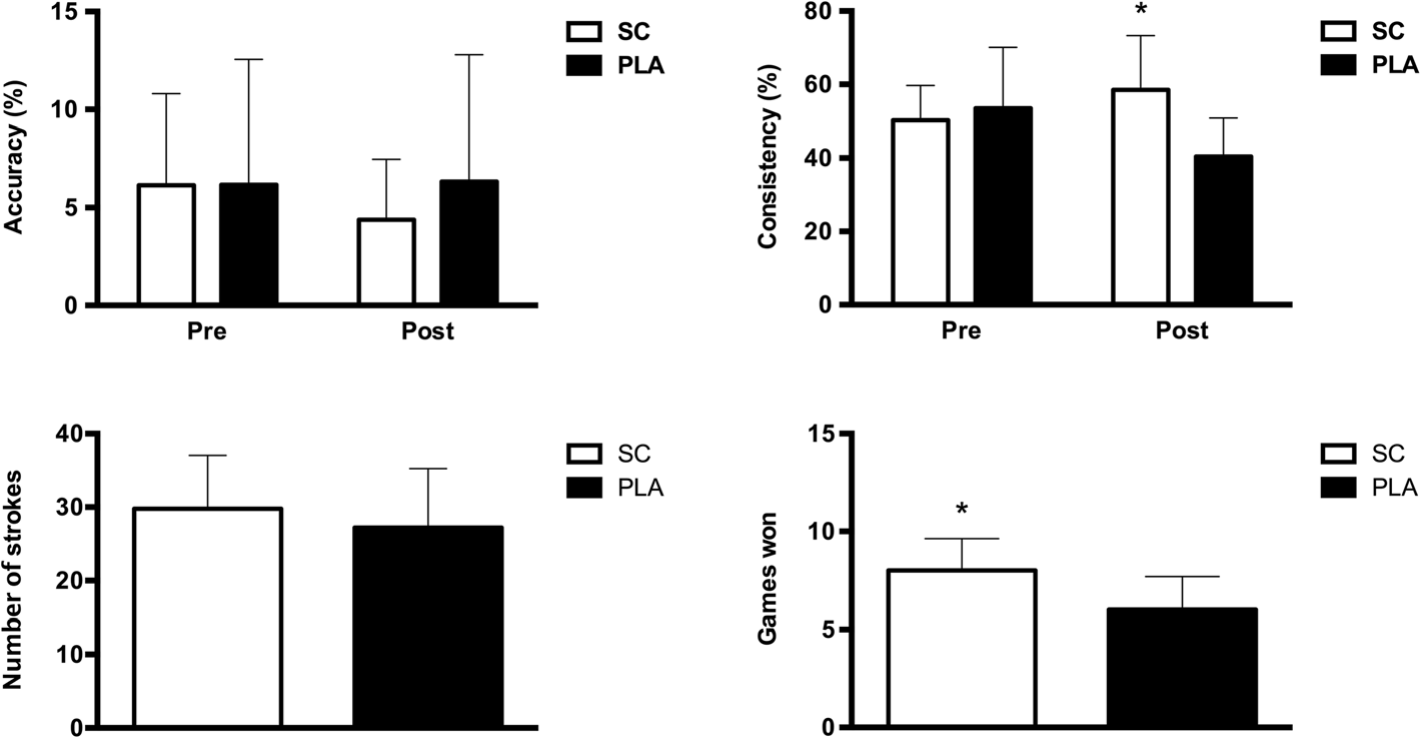
Data are mean ± standard deviation. Accuracy (%), consistency (%), number of strokes and games won in both experimental conditions. * different from Placebo (PLA)

A bar graph depiction of results for the STPT and games won during the simulated match can be seen in Fig. 1. Regarding the STPT, there was no difference (*p* > 0.05) in percentage shot accuracy or percentage shot consistency at the pre-match testing time point between PLA and SC. Further, no difference existed (*p* > 0.05) between conditions in shot accuracy or total strokes between conditions as post-match. However, SC did show a significantly greater (*p* < 0.01) percentage shot consistency at post-match compared to PLA (SC: 58.5 ± 14.8% vs. PLA: 40.4 ± 10.4%).

**Fig. 2:**
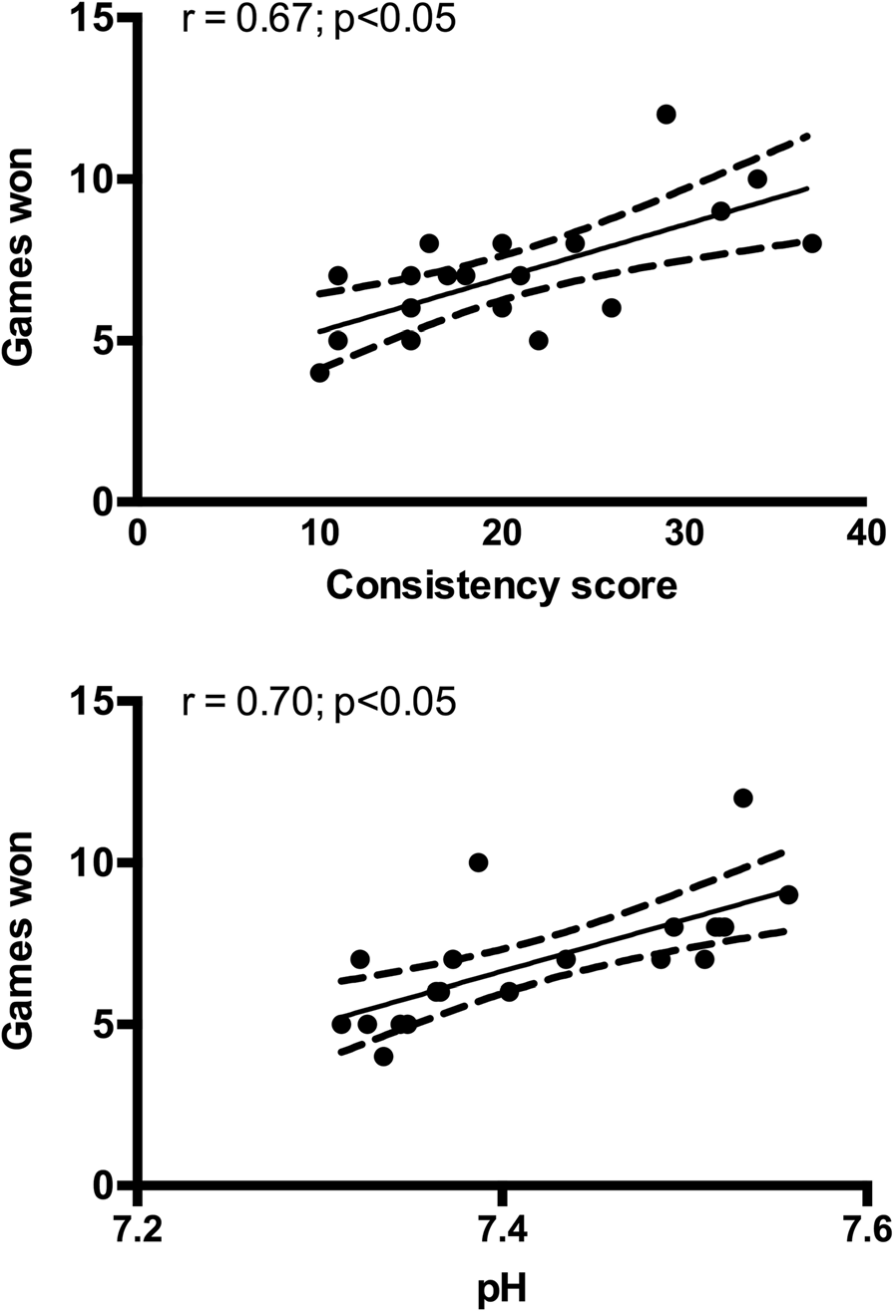
Correlation analysis between games won and post-match consistency score and games won and post-match pH

In SC, players won a significantly greater (*p* < 0.001) amount of games compared to PLA. Additionally, in SC there was a significant correlation between number of games won during the simulated match with both post-match percentage shot consistency (r = 0.67, p < 0.001) and post-match pH (r = 0.70, *p* < 0.0005) (Fig. 2). There was no difference in RSA performance from pre- to post-match for PLA (5.368 ± 0.413 to 5.205 ± 0.484 s; *p* > 0.05) or SC (5.342 ± 0.400 to 5.280 ± 0.382 s; *p* > 0.05), nor was there any difference between conditions (*p* > 0.05). Between conditions, ES for RSA at pre-match was 0.06, while ES at post-match was 0.17. For RSA there was also an average decrement from the first to the 10th sprint of 4.76% in performance in PLA (1st: 5.441 ± 0.470 to 10th: 5.713 ± 0.284). However, a decrement of only 0.1% occurred in SC from the first to the 10th sprint (1st: 5.371 ± 0.460 to 10th: 5.424 ± 0.322).

### RPE and GI questionnaire

No difference (*p* > 0.05) was observed in total session RPE score between SC (6.4 ± 1.2) and PLA (6.7 ± 1.8) conditions. In terms of the GI questionnaire only 3 subjects in SC reported mild symptoms of discomfort (i.e. abdominal pain, epigastric pain, abdominal noises, bloating, urge to burp, loss of appetite and flatulence), and only 2 subjects in SC noted a mild headache, while no subjects reported sever GI distress symptoms. No subjects reported GI discomfort following PLA.

### Metabolic parameters

**Fig. 3:**
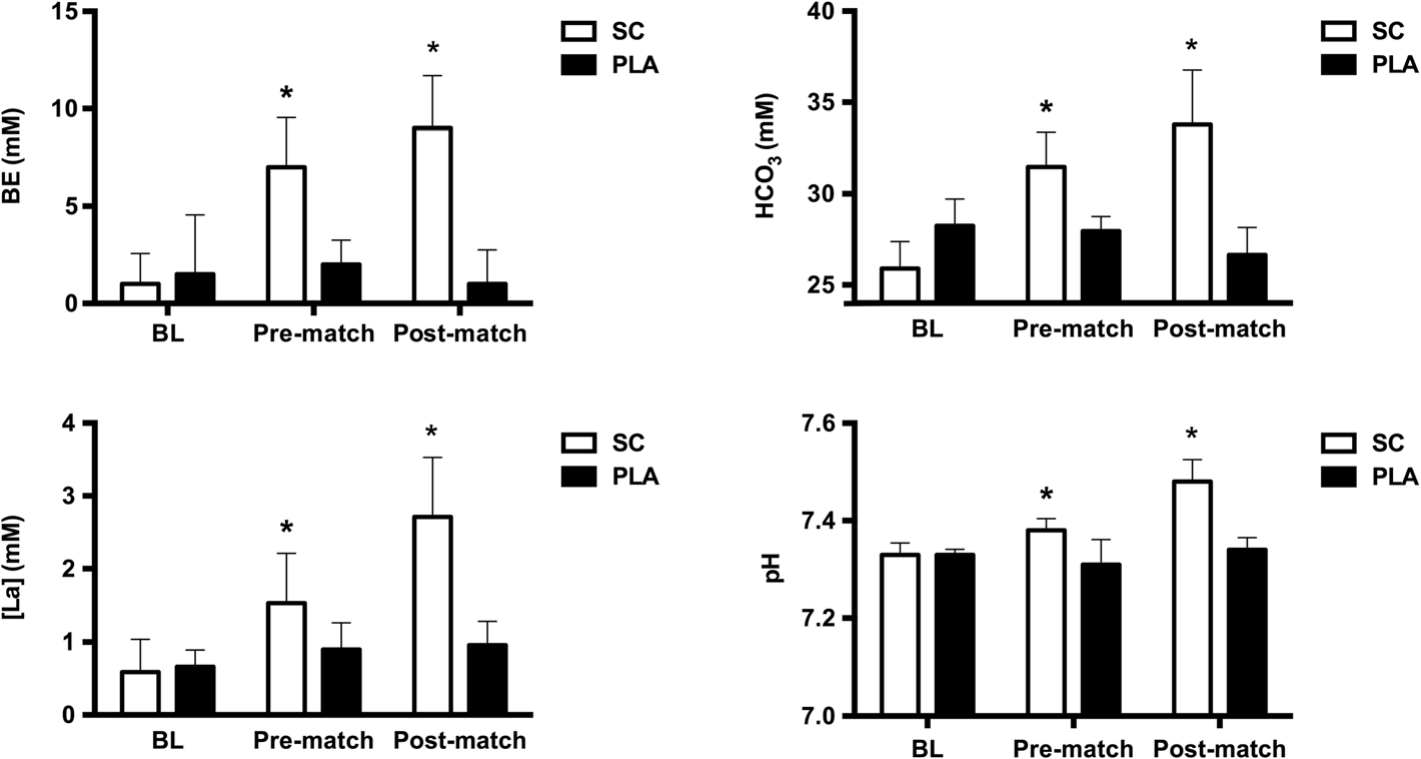
Metabolic parameters Data are mean ± standard deviation. Base excess (BE), bicarbonate (HCO_3_), lactate concentration ([La]) and pH level at baseline (BL), pre-match and post-match in both conditions. **a** - Greater than Placebo (PLA). **b** - Greater than Baseline (BL)

There was no difference (*p* > 0.05) in the level of any metabolic parameter at BL between conditions (Fig. 3) and BE, pH, and HCO_3_^−^ significantly increased (*p* < 0.05) from BL to pre-match in SC. All 4 metabolic markers showed an increase from BL to post-match in SC (*p* < 0.05), while there was no significant change (*p* > 0.05) from BL to pre- or post-match in any metabolic parameter in PLA. Between conditions, there was a statistically greater level at both pre- and post-match for BE (Pre, *p* < 0.001; Post, *p* < 0.001), pH (Pre, *p* < 0.001; Post, *p* < 0.001), HCO_3_^−^, and [La] (Pre,* p* < 0.05; Post, *p* < 0.001) in SC vs. PLA. Effect sizes demonstrated a large effect (> 0.80) in favor of SC for every metabolic parameter at both pre- and post-match. Percentage changes from BL to pre- and post-match of the metabolic parameters in SC were as follows: BE: 1.30 ± 1.57 to 6.30 ± 2.69 mM at pre-match (+ 384.62%) and 9.10 ± 2.69 mM at post-match (+ 600%), pH: 7.33 ± 0.02 to 7.38 ± 0.020 at pre-match (+ 0.68%) and 7.48 ± 0.05 at post-match (+ 2.05%), HCO_3_^−^: 25.90 ± 1.49 to 31.48 ± 2.00 mM at pre-match (+ 21.54%) and 33.78 ± 2.99 mM at post-match (+ 0.42%), [La]: 1.62 ± 1.06 to 2.54 ± 0.31 at pre-match (+ 56.79%) and 5.46 ± 1.11 mM at post-match (+ 237.04%).

## Discussion

The primary aim of the present study was to examine changes in metabolic parameters, tennis skill performance, and the perceptual response following SC supplementation or PLA in nationally ranked young Brazilian tennis players. The main findings of this investigation supported the hypothesis the SC would induce alkalosis and benefit performance. These findings were: 1) All metabolic parameters (i.e. BE, pH, HCO_3_^−^, and [La]) increased from BL to both pre- and post-match in SC, 2) The level of all metabolic parameters in SC compared to PLA at both pre- and post-match, 3) The SC condition resulted in a greater performance vs. PLA in terms of percentage shot consistency during the STPT and more games won during the simulated match, and 4) In SC, games won during the simulated match was positively correlated with both percentage shot consistency and pH.

It has been suggested that when [La] reaches up to 7-8 mM, tennis performance declines [5]. However, [La] more commonly increases to only 2-4 mM during match play [2, 18, 23]. Girard and Millet have suggested that elevated blood lactate may lead to mistimed strokes [18]. Presently, it was observed [La] concentration in SC to reach 5.46 ± 1.11 mM at post-match, which is lower than the upper end previously observed, however, the only 1-h of match time in the present study likely accounts for this lower level. Although a relationship between acidity and mistimed strokes was not observed presently, a positive relationship between pH and games won (r = 0.70) was observed suggesting that inducing alkalosis may enhance tennis performance.

This study showed a significantly greater level of BE, pH, HCO_3_^−^, and [La] in the players at post-match in SC compared to the PLA condition. Interestingly, the increase in [La] in SC occurred despite an increase in pH. However, a concomitant increase [La] and extracellular pH is in agreement with previous findings in which alkalosis has been induced [44–46]. Specifically, Wu et al. [23] induced alkalosis via NaHCO_3_ in male collegiate tennis players and still observed increased [La] just as the present study. Further, Stephens et al. [46] induced alkalosis via NaHCO_3_ during endurance cycling and concomitantly observed increased [La]. Mechanistically, the increased alkalosis observed in these studies is due to an increased H^+^ gradient leading to higher H^+^ and lactate expulsion from the working skeletal muscles due to monocarboxylate co-transporter (a carrier of H^+^ and lactate), which explains increased blood [La] in the presence of alkalosis [46]. Additionally, all metabolic parameters experienced a non-significant increase from pre- to post-match, which seems incongruent with fatigue that may have been caused by the simulated match. However, post-match blood collection occurred 3 h following supplementation, which is the duration (120 min) that Potteiger et al. [47] have previously demonstrated HCO_3_^−^ level to peak.

Fatigue has been explained as a reduction in maximal force capabilities of a muscle over time during exercise [48], thus delaying fatigue can maintain neuromuscular capabilites and potentially performance. Indeed, the current study not only noted superior shot consistency (%) in STPT and a greater amount of games won in the simulated match in SC compared to PLA, but also a positive correlation (r = 0.70) between pH level and games won. Therefore, it seems that SC was able to prolong muscle contractile capabilities. Theoretically, prolonging muscle force production would enhance an athlete’s ability to perform training volume, which is the training variable most closely and positively associated with muscle performance adaptations [49].

Interestingly, the present investigation did not report a significant difference (*p* > 0.05) between session RPE in SC (6.45 ± 1.21) vs. PLA (6.7 ± 1.84) despite the improved metabolic profile and performance in SC. However, this finding is in agreement with previous data, which has induced alkalosis via NaHCO_3,_ and noted specific skill performance increase in tennis [23] and boxing [50], but no difference between experimental and control conditions in terms of session RPE. Thus, the perceptual response may not be related to specific skill performance.

The dosage of SC in this study was based off of McNaughton and Cedaro [8], which demonstrated 0.5 g^.^kg^−1^BM of SC significantly increased buffering capacity, total work, and peak power during anaerobic cycling performance of durations of 120 s and 240 s in healthy males. Additionally, McNaughton [28] reported a performance enhancement after SC supplementation in a 1 min maximal cycle ergometer test; however, Cox and Jenkins [30] did not find SC supplementation to improve performance in repeated 60 s cycling sprint performance despite inducement of alkalosis. Furthermore, McNaughton and Cedaro [8] did not observe a performance benefit in cycling of a 10 s duration, which may explain the lack of difference in RSA times between SC and PLA in the present study; as each RSA test lasted approximately 5 s. It has been suggested that metabolic factors may not be of great consequence in such a short trials [26], since reliance on glycolysis is decreased and buffering H^+^ is not of great consequence in this short duration. However, Carr et al. [31] concluding in a meta-analysis that NaHCO_3_ improves 1 min sprint performance by 1.7% and has compared favorably to SC for running sprint performance lasting approximately 80s. To explain the differential findings between SC and NaHCO_3_ for sprint performance, Van Montfoort et al. [51] noted that despite SC inducing extracellular alkalosis the intracellular increase in citrate may blunt ATP resynthesis, whereas NaHCO_3_ would enhance ATP resynthesis. This explanation may account for the varying results between the 2 alkalizing agents and the lack of benefit of SC for RSA performance in the present study.

Even though NaHCO_3_ has been an effective buffer a possible drawback is the possible GI distress. Importantly, the GI distress with both NaHCO_3_ and SC is equivocal [26]. The current results for GI distress revealed that only 3 subjects reported even mild symptoms of GI discomfort (i.e. abdominal pain epigastric pain, abdominal noises, bloating, urge to burp, loss of appetite and flatulence), and only 2 subjects acknowledged a mild headache with no reports of severe GI discomfort following the SC condition. Thus, although NaHCO_3_ and SC may both be effective as extracellular buffers, SC may be the more attractive option in an effort to avoid GI discomfort. However, more research should be conducted with NaHCO_3_ in tennis players, in which GI discomfort is specifically monitored.

A possible limitation to this study was the small sample size. However, a previous investigation [23], also in male tennis players, used only 9 subjects (one fewer than the present study) to examine NaHCO_3_ supplementation in a double blind fashion. Further, this study is unique as it is the first study to measure specific tennis skills as an outcome measure in response to SC supplementation, adding novelty to the investigation.

## Conclusion

In conclusion, SC supplementation (0.5 g^.^kg^−1^BM in capsules of 500 mg) significantly altered metabolic parameters (i.e. BE, pH, HCO_3_^−^, [La]) and improved skilled tennis performance (i.e. percent shot consistency and games won during simulated match play) compared to PLA. Importantly, no players reported any significant GI discomfort following SC supplementation. Practically, it appears that SC supplementation can be safely and effectively utilized in tennis players to enhanced skilled performance. Although, it cannot be known from these results if SC supplementation would be beneficial in long lasting matches, previous data has shown SC to be effective for up to 30 km of cycling, thus it is possible that SC could be beneficial in matches lasting for multiple hours. Importantly, if SC is utilized in training sessions, training volume could be increased to allow for greater duration of quality skill practice. Additionally, it should be noted that individual athletes may have varying GI responses to SC, even though no athletes reported discomfort in the present study, thus future studies should continue to investigate the efficacy/safety of SC supplementation in various athletic populations.

## Data Availability

The datasets generated during and/or analysed during the current study are available from the corresponding author on reasonable request.
